# Osteotomy in “Genu Valgum”

**Published:** 1877-03

**Authors:** 


Osteotomy in "Genu Valgum."—At the Congress of Naturalists recently held in Hamburg, an interesting paper on this subject was read in the Surgical Section, by Dr. Max Schede. Antiseptists will be pleased to know that the successful results, to which we will shortly refer, were considered in great measure as due to the antiseptic precautions and dressings which were adopted in the cases. Dr. Max Schede presented to the Congress a young man on whom he had performed cuneiform osteotomy of both tibiæ. The man had been the subject of exaggerated *genu valgum*, which had resisted all previous treatment. The legs and thighs were at an angle of about eighty degrees. Antiseptic precautions were adopted at the operation (which was done in February), and Esmarch's bandage was applied. A wedge-shaped piece of bone, three-quarters of an inch wide, was excised from each tibia, and the fibula was cut across; the legs were then straightened. At the end of May, consolidation was complete, and the operation thoroughly successful. At the same time he reported other cases where the operation had been done with similar success either for rickets or knocknees. He mentioned one case in which the tibia had been divided but not the fibula, but the cure was not so complete as in those cases where both bones had been cut. Mr. Howard Marsh recently read before the Royal Medical and Chirurgical Society a paper on Osteotomy of the Tibia for Deformities caused by Rickets; he referred to some successful cases in which he had performed this operation at the Children's Hospital in Great Ormond Street. When we consider how helpless the subjects of extensive rickets and severe knock-knees may become, it is a matter for congratulation to think that surgeons are now able to tackle these diseases with such good chances of success, and with so little risk to the patient.—*Med. Times and Gaz.*, Jan. 6, 1877.

